# A Regulatory Element in the Intrinsically Disordered C-Terminal Region of LMTK3 Modulates Its Kinase Domain Interactions and Breast Cancer Phenotypes

**DOI:** 10.3390/cells15161473

**Published:** 2026-08-17

**Authors:** Andrea Lauer Betrán, Alessandro Agnarelli, Mark Samuels, Viviana Vella, Reza Shirazi Nia, Daniel De Vega, Niloufar Poudine, Daniela Carter-Lopez, Angeliki Ditsiou, Murat Eravci, Chrisostomos Prodromou, Erika J. Mancini, Georgios Giamas

**Affiliations:** 1 Department of Biochemistry and Biomedicine, School of Life Sciences, University of Sussex, Falmer, Brighton BN1 9QG, UK; a.p.lauer-betran@sussex.ac.uk (A.L.B.); a.agnarelli@qmul.ac.uk (A.A.); mark.t.samuels@gmail.com (M.S.); vv62@sussex.ac.uk (V.V.); dsc48@cam.ac.uk (D.C.-L.); a.ditsiou@sussex.ac.uk (A.D.); eravci136@gmail.com (M.E.); chris.prodromou@sussex.ac.uk (C.P.); 2 Centre for Cancer Cell & Molecular Biology, Barts Cancer Institute, Queen Mary University of London, London EC1M 6BQ, UK; 3 International Oncology Institute, The First Affiliated Hospital of Zhejiang Chinese Medical University, Hangzhou 310053, China; rezashirazinia@zcmu.edu.cn (R.S.N.); danieldevega@zcmu.edu.cn (D.D.V.); niloufarpd@gmail.com (N.P.); 4 Department of Pathology, School of the Biological Sciences, University of Cambridge, Cambridge CB2 0QQ, UK; 5 Sussex Drug Discovery Centre, School of Life Sciences, University of Sussex, Falmer, Brighton BN1 9QG, UK; erika.mancini@sussex.ac.uk

**Keywords:** LMTK3, kinase regulation, intrinsically disordered region, phosphorylation, breast cancer

## Abstract

Lemur tail kinase 3 (LMTK3) is an oncogenic Ser/Thr kinase implicated in breast cancer (BC) progression, therapy resistance, and poor clinical outcomes, yet the molecular mechanisms governing its regulation remain poorly understood, particularly the role of its C-terminal intrinsically disordered region (IDR). Given that IDRs frequently harbour hidden structural motifs that control protein dynamics, we combined computational, biophysical, and biochemical approaches to systematically map regulatory elements within the LMTK3 C-terminus, identifying two regions (residues 688–1095 and 1181–1486) that interact with the LMTK3 kinase domain (LMTK3-KD). Characterisation of these interactions revealed that LMTK3^1181–1486^ displays preferential binding to inactive wild-type LMTK3-KD over a constitutively active mutant (LMTK3-KD^L313R^), a behaviour consistent with a potential autoinhibitory interaction. Guided by AlphaFold3 modelling, we localised this interaction primarily to a short α-helical motif (α-helix 2; residues 1247–1258) within the C-terminal IDR and subsequently identified Ser1258 within this motif as a candidate regulatory phosphorylation site, using [γ-^32^P]-ATP kinase assays and mass spectrometry. Phosphorylation at Ser1258 altered interactions between α-helix 2 and the kinase domain, reducing binding to wild-type LMTK3-KD while increasing affinity for LMTK3-KD^L313R^. Functionally, phospho-null mutation of Ser1258 impaired oestrogen receptor alpha (ERα) upregulation, proliferation, migration, and clonogenicity in ER-positive BC cell lines, and reduced tumour growth in female BALB/c nude mice bearing orthotopic MCF7 xenografts. Together, these findings identify a previously uncharacterised regulatory element within the LMTK3 C-terminus and support a model in which Ser1258 phosphorylation modulates kinase domain interactions and LMTK3-driven oncogenic functions in BC.

## 1. Introduction

Protein kinases are central regulators of cellular signalling, controlling processes including metabolism, apoptosis, proliferation, differentiation, and cell cycle progression through substrate phosphorylation [1,2,3,4,5]. Given that aberrant kinase activity is a major driver of oncogenesis, these enzymes have emerged as key drug targets [6,7]. Accordingly, understanding the molecular mechanisms that regulate kinase activity is essential for the rational development of next-generation therapeutics.

Kinase regulation commonly involves transitions between active and inactive conformational states driven by coordinated movements of the αC-helix, activation loop (A-loop), and Asp-Phe-Gly (DFG) motif [8,9,10,11]. In the active conformation, the extended A-loop configuration allows the Asp residue of the DFG motif to coordinate Mg^2+^ ions, thereby positioning ATP for catalysis [12]. This state is further stabilised by a conserved salt bridge between the αC-helix Glu and the β3-strand Lys, promoting alignment of key residues that enable phosphate transfer. Conversely, many inactive conformations are characterised by reorientation of the DFG motif, disrupting the active-site architecture required for kinase activity [13]. These conformational states also influence pharmacological susceptibility: type I inhibitors preferentially bind open, active conformations whereas type II inhibitors bind inactive states [12,14]. Beyond conformational switching, phosphorylation, autoinhibition, allosteric interactions, and subcellular localisation provide further spatial and temporal control of kinase activity [15,16,17,18,19,20,21,22,23,24]. Canonical examples include the SH2/SH3-mediated autoinhibition in Src family kinases [25,26,27] and ligand-induced dimerisation of the epidermal growth factor receptor (EGFR), which promotes allosteric activation of the kinase domain [28,29,30].

Despite significant advances in kinase biology [12], a substantial fraction of the human kinome remains poorly characterised, the so-called *dark kinome*, which includes the lemur tail kinases (LMTKs) [31]. All three members of the LMTK family (LMTK1–3) share a conserved kinase domain and an extended C-terminal tail enriched in Pro, Ser, and Gly residues that is predicted to be intrinsically disordered [32,33,34,35]. LMTK3, in particular, has attracted considerable attention due to its frequent overexpression in breast cancer (BC), where it correlates with poor overall and disease-free patient survival [36,37]. Functional studies have implicated LMTK3 as an oncogenic driver in BC, with reported roles in oestrogen receptor alpha (ERα) stabilisation, invasion, metastasis, and therapy resistance [33,38,39,40,41,42,43,44,45,46]. Elevated LMTK3 expression has also been observed across multiple cancer types, suggesting broader oncogenic potential [47,48,49,50,51,52,53]. In addition to its role in cancer, LMTK3 has been implicated in central nervous system function, with roles in behaviour, cognition, and synaptic regulation [54,55,56,57]. More recently, X-ray crystallographic studies have resolved the atomic structure of the inactive LMTK3 kinase domain (LMTK3-KD), spurring the development of targeted small-molecule inhibitors with demonstrated antitumour activity in preclinical models [41,58]. While these advances have provided valuable insight into the biological and therapeutic relevance of LMTK3, the molecular mechanisms regulating its function remain poorly understood. In particular, the role of its extended intrinsically disordered C-terminal tail–a defining yet largely unexplored feature of the LMTK family–remains unclear [32,33,34].

In this study, we used integrated computational, biophysical, biochemical, cellular, and *in vivo* approaches to investigate structural and functional regulatory elements within the LMTK3 intrinsically disordered C-terminus. We mapped direct intramolecular interactions between the LMTK3-KD and two distinct C-terminal segments (residues 688–1095 and 1181–1486) and further characterised a short α-helical motif within the latter (α-helix 2; residues 1247–1258) as a putative interaction site involved in binding to LMTK3-KD. Furthermore, we identified Ser1258 (S1258) within this motif as a potential phosphorylation site *in vitro* and showed that phosphorylation at this site operates as a regulatory switch, reversing the motif’s binding preference from the inactive to the active kinase state. Finally, we established the functional relevance of this regulatory switch by demonstrating that the phospho-null S1258A mutation impairs ERα stabilisation, cell proliferation, and migration in ER-positive BC cell lines, while also significantly suppressing tumour growth in female BALB/c nude mice bearing orthotopic MCF7 xenografts. Global quantitative TMT-proteomic and phosphoproteomic profiling further revealed that disrupting the S1258 switch remodels essential cell-cycle, DNA replication, and mitotic progression machinery, including core components of the replication-essential GINS complex. Together, our findings identify a previously uncharacterised regulatory element within the LMTK3 C-terminal tail and provide new insights into the molecular mechanisms underlying LMTK3-dependent BC progression.

## 2. Materials and Methods

### 2.1. Cell Lines and Culture Conditions

T47D and MCF7 BC cell lines were obtained from the American Type Culture Collection (ATCC, Manassas, VA, USA, cat. nos. HTB-133 and HTB-22, respectively). MCF7 cells were cultured in low-glucose Dulbecco’s Modified Eagle Medium (DMEM, Sigma Aldrich, St. Louis, MO, USA, cat. no. D6046) supplemented with 10% foetal bovine serum (FBS, Sigma Aldrich, cat. no. F7524) and 1% penicillin/streptomycin (P/S, Sigma Aldrich, cat. no. P0781). T47D cells were maintained in Roswell Park Memorial Institute medium (RPMI-1640, Sigma Aldrich, cat. no. R5886) supplemented with 10% FBS and 1% P/S solution. Cells were cultured in a humidified incubator at 37 °C with 5% CO_2_ and were routinely tested for mycoplasma contamination.

### 2.2. Generation of Stable Cell Lines

Cells were seeded at 2.5 × 10^5^ cells/well in 6-well plates using antibiotic-free medium. Cells were transfected with episomal S/MAR plasmids [59,60] encoding full-length wild-type (WT) LMTK3 or a phospho-null (S1258A) mutant using FuGENE 4K transfection reagent (Promega, Madison, WI, USA, cat. no. E5911) according to the manufacturer’s instructions. An episomal S/MAR empty vector (EV) was used as a control. At 48 h post-transfection, cells were selected with 800 μg/mL Geneticin (G418 Sulphate, Thermo Fisher, Grand Island, NY, USA, cat. no. 10131035) for three weeks. To isolate single-cell clones, resistant colonies were detached, passed through a 40 μm cell strainer, and seeded into 96-well plates at one cell per well. Clones exhibiting comparable LMTK3 expression levels were selected for downstream experiments, with EV clones serving as negative controls.

### 2.3. Cell Proliferation Assay

Cells were seeded in 96-well plates at 3 × 10^3^ cells/well and incubated for 96 h at 37 °C. Cells were fixed with 4% paraformaldehyde (PFA, Sigma Aldrich, cat. no. P6148) prepared in Dulbecco’s Phosphate-Buffered Saline (DPBS, Sigma Aldrich, cat. no. D8537) and stained with 0.1% crystal violet (Thermo Fisher, cat. no. B21932.14) dissolved in 10% ethanol. Unbound dye was removed by thorough washing with DPBS, and plates were air-dried. Bound dye was solubilised with 10% acetic acid under gentle agitation, and absorbance was measured at 590 nm using a GloMax-Multi detection system (Promega). Values from six technical replicates per condition were averaged, normalised to the EV control, and expressed as percentages. Data represent three independent biological replicates (*n* = 3).

### 2.4. Wound Healing Assay

Cells were seeded in 24-well plates at 3 × 10^5^ cells/well in serum-free medium to establish confluent monolayers. Monolayers were scratched manually using a sterile 10 μL pipette tip, washed with DPBS to remove detached cells, and maintained in serum-free medium throughout the assay. Images were captured at 0 h and 24 h post-scratch using a JuLI Stage Real-Time Cell History Recorder (NanoEntek, Hwaseong-si, Gyeonggi-do, Republic of Korea). Wound area was quantified using ImageJ v1.53 (U.S. National Institutes of Health, Bethesda, MD, USA) by manually tracing the leading edges of the cell fronts and measuring the cell-free area. Values from three technical replicates per condition were averaged at each time point, and percentage wound closure was calculated as:

$$\%\;Wound\;closure=(1-\frac{\left(wound\;area\;at\;24\;h\right)}{\left(wound\;area\;at\;0\;h\right)})\times 100$$

Data represent three independent biological replicates (*n* = 3).

### 2.5. Clonogenic Assay

Cells were seeded in 6-well plates at 50 cells/well and incubated until colonies contained at least 50 cells, in accordance with established guidelines [61]. Colonies were fixed with 4% PFA in DPBS and stained with 0.1% crystal violet in 10% ethanol. Colonies were manually counted, and only well-defined, rounded colonies with dense morphology were included in the analysis. Data represent three independent biological replicates (*n* = 3).

### 2.6. Western Blot Analysis

Cells were lysed on ice for 1 h in radioimmunoprecipitation assay (RIPA) buffer (Sigma Aldrich, cat. no. R0278) supplemented with protease and phosphatase inhibitors (Roche Diagnostics, Basel, Switzerland, cat. nos. 11697498001 and 4906845001, respectively). Lysates were clarified by centrifugation at 16,000× *g* for 30 min at 4 °C, and protein concentration was determined using a BCA Protein Assay Kit (Thermo Fisher, cat. no. 23227). 40 µg of protein was resolved by SDS-PAGE and transferred to 0.2 µm nitrocellulose membranes (Thermo Fisher, cat. no. IB23001) using an iBlot 2 device (Thermo Fisher). Membranes were blocked for 1 h at room temperature in Tris-buffered saline (TBS) containing 0.1% Tween-20 and 5% non-fat dry milk, followed by overnight incubation at 4 °C with primary antibodies against α-tubulin (GenScript, Piscataway, NJ, USA, cat. no. A01410), ERα (Cell Signalling, Danvers, MA, USA, cat. no. 8644), or LMTK3 (Sigma Aldrich, cat. no. WH0114783M2). Horseradish peroxidase (HRP)-conjugated secondary antibodies (Cell Signalling; anti-mouse cat. no. 7076; anti-rabbit cat. no. 7074) were applied, and signals were detected using SuperSignal West Pico PLUS (Thermo Fisher, cat. no. 34580) on a Odyssey FX imaging system (LI-COR Biosciences, Lincoln, NE, USA). Densitometric analysis was performed using ImageJ. Band intensities were normalised to loading controls and subsequently to the EV control. Data represent three independent biological replicates (*n* = 3).

### 2.7. Cell-Based Global Proteomic and Phosphoproteomic Analysis

T47D cell pellets from four independent biological replicates per condition were resuspended in RIPA buffer supplemented with Halt protease and phosphatase inhibitors (Thermo Fisher, cat. no. 78440). Samples were incubated on ice for 15 min with intermittent vortexing, sonicated for 10 s, and centrifuged at 14,000× *g* for 15 min. Protein concentration was determined using a BCA Protein Assay Kit. For each sample, 100 µg of protein was digested overnight with trypsin at a 1:40 enzyme-to-protein ratio. Resulting peptides were labelled using the TMTpro 16-plex Label Reagent Set (Thermo Fisher, cat. no. A44520) according to the manufacturer’s instructions, and all labelled samples were then pooled. A 50 µg aliquot of the pooled TMT-labelled sample was desalted using Sep-Pak cartridges (Waters, Milford, MA, USA, cat. no. WAT051910). Peptides were dried, resuspended in 20 mM ammonium hydroxide (pH 10), and fractionated by high-pH reversed-phase chromatography using an Ultimate 3000 system (Thermo Fisher) equipped with an XBridge BEH C18column (Waters). Peptides were eluted using a 0–95% gradient of 20 mM ammonium hydroxide in acetonitrile (pH 10) over 60 min, dried, and then resuspended in 1% formic acid. The remaining pooled TMT-labelled sample was desalted using Sep-Pak cartridges, dried, and subjected to phosphopeptide enrichment using TiO_2_ affinity chromatography (Thermo Fisher, cat. no. 88301). Flow-through fractions were further enriched using Fe-NTA chromatography (Thermo Fisher, cat. no. A32992), dried, and resuspended in 1% formic acid. Both high-pH fractionated peptides and phosphopeptide-enriched samples were analysed by liquid chromatography-tandem mass spectrometry (LC-MS/MS) using an Ultimate 3000 nanoLC system coupled to an Orbitrap Fusion Lumos mass spectrometer (Thermo Fisher). Peptides were loaded onto an Acclaim PepMap C18 analytical column (Thermo Fisher) and separated with a 150 min acetonitrile gradient (1–90%) in 0.1% formic acid at 300 nL/min. Peptides were ionised by nano-electrospray ionisation at 2.0 kV using a stainless-steel emitter (30 μm internal diameter; Thermo Fisher) with a capillary temperature of 300 °C. Data were acquired in data-dependent acquisition (DDA) mode, with FTMS1 spectra recorded at 120,000 resolution using an automatic gain control (AGC) target of 200,000 and a maximum injection time of 50 ms. Dynamic exclusion was applied for 60 s (±10 ppm), and MS2 precursor ions were isolated within a 0.7 m/z window. ITMS2 spectra were acquired with an AGC target of 10,000 and a maximum injection time of 70 ms. For FTMS3 analysis, the Orbitrap was operated at 50,000 resolution with an AGC target of 50,000 and a maximum injection time of 105 ms. Precursor ions were fragmented by higher-energy collisional dissociation (HCD) at a normalised collision energy of 60%. Raw data were processed using Proteome Discoverer v2.4 (Thermo Fisher) and searched against the SwissProt human database (downloaded February 2026; 26,274 entries), supplemented with WT LMTK3 and LMTK3 S1258A mutant sequences, using the SEQUEST HT algorithm. Precursor mass and MS/MS tolerances were set to 10 ppm and 0.6 Da, respectively. Carbamidomethylation of Cys and TMTpro modification of peptide N-termini and Lys residues were set as fixed modifications. Variable modifications included Met oxidation, N-terminal acetylation, Met loss from the protein N-terminus, and Met loss plus N-terminal acetylation. For phosphoproteomic analysis, phosphorylation of Ser, Thr, and Tyr was also included as a variable modification. Up to two missed tryptic cleavages were permitted, and peptide-spectrum matches were filtered to a 5% false discovery rate (FDR). Differential expression analysis was performed using the limma package [62]. A single linear model was fitted across all conditions, and moderated *t*-tests were used for comparisons of interest. *p*-values were adjusted for multiple testing using the Benjamini–Hochberg procedure. Enrichment analysis was performed using Ingenuity Pathway Analysis (IPA, QIAGEN, Hilden, Germany) [63]. Core expression and phosphorylation analyses were conducted on total proteome and phosphoproteome datasets, respectively, using all quantified proteins as the reference set. Statistical significance for enrichment analyses was defined as FDR < 0.01 (Benjamini–Hochberg adj. *p*-values).

### 2.8. Animal Experiments

Orthotopic xenograft studies were performed using twenty specific pathogen-free (SPF) female BALB/c nu/nu athymic nude mice (4–5 weeks old; Shanghai SLAC Experimental Animal Co., Ltd., Shanghai, China). All animal procedures were approved by the Institutional Animal Care and Use Committee of Zhejiang Chinese Medical University (Hangzhou, China; Approval no: IACUC-20241216-23) and conducted in accordance with institutional guidelines. Upon arrival, mice were confirmed to be healthy by veterinary assessment, acclimated for one week, and housed under SPF conditions (five mice per cage) on corncob bedding at 24–26 °C with 40–70% relative humidity under a 12 h light/dark cycle, with *ad libitum* access to standard pelleted chow and water. Mice were randomly assigned to receive orthotopic injections of MCF7 cells stably overexpressing either WT LMTK3 or the LMTK3 S1258A mutant (*n* = 10 per group) using body weight-stratified randomisation to ensure balanced baseline body weights between groups. Sample size was determined based on previous experience with the orthotopic MCF7 xenograft model while adhering to the principles of the 3Rs to minimise animal use. To support tumour establishment, mice received subcutaneous injections of 17β-oestradiol (20 µg in 20 µL; MedChemExpress, Monmouth Junction, NJ, USA, cat. no. HY-B0141) prepared in a 9:1 corn oil:ethanol vehicle, beginning three days before tumour cell inoculation and continuing five times per week throughout the experiment. Under isoflurane anaesthesia (2.5% for induction and 2% for maintenance), 5 × 10^6^ cells were suspended in a 1:1 mixture of DMEM and Matrigel (Corning, Corning, NY, USA, cat. no. 356231) and injected into the fourth mammary fat pad. Tumour dimensions were measured three times weekly using digital callipers, and tumour volume was calculated as:

$$Volume=\frac{\left(width^{2}\times length\right)}{2}$$

When tumours reached approximately 60–70 mm^3^, this was designated as Day 1 of the study. Tumour growth and body weight were then monitored for a further 21 days until the planned study endpoint. Animal welfare was monitored throughout according to predefined humane endpoint criteria, including tumour volume ≥ 1000 mm^3^, body weight loss ≥ 25%, cachexia, tumour ulceration, anorexia lasting more than 24 h, or evidence of severe organ dysfunction or distress. One mouse in the WT LMTK3 group reached a predefined humane endpoint on the final study day and was euthanised immediately after tumour measurement. No data were excluded from the analysis.

### 2.9. Immunohistochemistry

Paraffin-embedded tumour tissues were sectioned at 8 μm and mounted on poly-L-lysine-coated slides. Sections were deparaffinised at 60 °C for 10 min, followed by two changes in xylene and rehydration through graded ethanol (100%, 95%, 80%, and 50%), with final washes in phosphate-buffered saline (PBS). Antigen retrieval was performed in 10 mM citric acid (pH 6.0) at 120 °C for 30 min under pressure. After cooling, sections were washed in PBS and incubated with 0.3% hydrogen peroxide in PBS for 40 min. Sections were then washed in PBS containing 0.3% Triton X-100 (PBS-T) and blocked in 10% FBS and 1% Bovine Serum Albumin (BSA) in PBS-T for 1 h at room temperature. Sections were incubated overnight at 4 °C with Ki-67 primary antibody (Abcam, Cambridge, UK, cat. no. ab15580). The following day, sections were incubated with HRP-conjugated anti-rabbit secondary antibody (Cell Signalling, cat. no. 7074) for 1 h at room temperature. Immunoreactivity was visualised using 3,3′-diaminobenzidine (DAB) for 5 min. Sections were then dehydrated through graded ethanol, cleared in xylene, and mounted with DPX mounting medium (Sigma Aldrich, cat. no. 06522). Quantification was performed using ImageJ with colour deconvolution (H-DAB) to separate DAB staining from haematoxylin. Twelve tumour samples (*n* = 6 per group) were analysed, with one representative region quantified per sample. Positive nuclei were quantified as a percentage of total nuclei.

### 2.10. LMTK3 Protein Expression and Purification

Fragments of the human LMTK3 gene corresponding to residues 688–1095 or 1181–1486 (residue numbering throughout this manuscript refers to UniProt entry A0A3B3ISL5) were cloned into pET28a (Addgene, Watertown, MA, USA, cat. no. 26094) and transformed into *E. coli* Rosetta2 (DE3) cells (Novagen, Madison, WI, USA). Large-scale cultures were grown in Luria–Bertani (LB) medium at 37 °C with shaking at 200 rpm until OD_600_ reached 0.6, then induced with 0.5 mM isopropyl β-d-1-thiogalactopyranoside (IPTG, Thermo Fisher, cat. no. R0392) and incubated for 16 h at 18 °C. Cells were harvested by centrifugation and resuspended in lysis buffer [50 mM Tris-HCl pH 7.5, 300 mM NaCl, 5 mM imidazole, 0.5 mM tris(2-carboxyethyl)phosphine (TCEP, Sigma Aldrich, cat. no. C4706), 0.1% Tween-20], supplemented with protease inhibitors (Thermo Fisher, cat. no. A32965) and DNAse I (Thermo Fisher, cat. no. 90083). Cells were lysed by sonication on ice (2 min total, 5–10 s pulses at 35% amplitude), and lysates were clarified by centrifugation at 30,000× *g* for 45 min at 4 °C. Supernatants were applied to HisPur Cobalt resin (Thermo Fisher, cat. no. 89965) pre-equilibrated with wash buffer (50 mM Tris-HCl pH 7.5, 300 mM NaCl, 5 mM imidazole, 0.5 mM TCEP) and incubated at 4 °C for 30 min with rotation. After washing, proteins were eluted with 200 mM imidazole. Eluates were concentrated and further purified by size-exclusion chromatography at 4 °C using a Superdex 200 Increase 10/300 GL column (GE Healthcare, Chicago, IL, USA) pre-equilibrated in gel-filtration buffer (25 mM Tris-HCl pH 8, 300 mM NaCl, 0.5 mM TCEP). Fractions containing LMTK3 constructs were identified by SDS-PAGE, pooled, and concentrated. Protein identity was confirmed by in-gel digestion followed by LC-MS/MS and Western blotting using a 6×-His tag antibody (Invitrogen, Waltham, MA, USA, cat. no. MA1-21315). Expression and purification of phospho-mimetic (S1258D) and phospho-null (S1258A) LMTK3^1181–1486^ mutants were performed as described above. WT LMTK3-KD and constitutively active LMTK3-KD^L313R^ proteins were expressed and purified as previously reported [41].

### 2.11. Microscale Thermophoresis (MST)

Fluorescent labelling of LMTK3-KD (WT or L313R, 10 μM) was performed using the RED-NHS 2nd Generation Protein Labelling Kit (NanoTemper Technologies, Munich, Germany, cat. no. MO-L011) according to the manufacturer’s instructions. Ligands (LMTK3 C-terminal constructs or synthetic peptides) were prepared in a 16-step serial dilution in assay buffer (20 mM Tris-HCl pH 8, 300 mM NaCl, 0.5 mM TCEP, 0.1% Tween-20) and mixed with a fixed concentration of fluorescently labelled LMTK3-KD (Table 1). Samples were incubated at room temperature for 10 min and centrifuged at 16,000× *g* for 10 min prior to loading into standard treated glass capillaries (NanoTemper Technologies, cat. no. MO-K022). Thermophoresis measurements were performed using a Monolith NT.115 instrument (NanoTemper Technologies) at 18–22 °C, with 20–60% excitation and 60% MST power. At least three independent measurements were acquired for each interaction. Binding affinities were determined by fitting the data using a standard *K*_d_ model in MO Affinity Analysis software v2.2.4 (NanoTemper Technologies).

### 2.12. In Vitro Kinase Assay

Kinase reactions were prepared in 1× kinase buffer (Cell Signalling, cat. no. 9802) supplemented with 20 μM ATP (Cell Signalling, cat. no. 9804) and 2.5 μCi ATP radiolabelled with phosphorus-32 at the gamma phosphate ([γ-^32^P]-ATP, Hartmann Analytic, Braunschweig, Germany, cat. no. FP-301). Reactions were incubated at 30 °C for 1 h, unless otherwise stated, and then resolved by SDS-PAGE. Gels were stained overnight at room temperature, dried using a vacuum-heated gel dryer at 64 °C for 2 h, and exposed to X-ray film for autoradiography. Phosphorylation signals were quantified using ImageJ and normalised to total substrate protein [64]. Experiments were performed in triplicate using independently prepared protein samples.

### 2.13. In Vitro LC-MS/MS Analysis of LMTK3 Phosphorylation

*In vitro* kinase reactions were performed in duplicate using purified LMTK3^1181–1486^ (10 μg) and WT LMTK3-KD (5 μg), excluding [γ-^32^P]-ATP. Substrate-only reactions were included as negative controls. Proteins were separated by SDS-PAGE and visualised by Coomassie staining. Relevant gel bands were excised, destained in 25 mM ammonium bicarbonate/50% acetonitrile overnight, reduced with 10 mM dithiothreitol at 50 °C for 30 min, and alkylated with 55 mM iodoacetamide at room temperature for 20 min in the dark. In-gel digestion was performed overnight at 37 °C using trypsin at a 1:50 enzyme-to-protein ratio. Peptides were extracted using Pierce Peptide Desalting Spin Columns (Thermo Fisher, cat. no. 89852) and enriched for phosphopeptides using Fe-NTA affinity chromatography. Samples were dried, reconstituted in 1% formic acid, and analysed by nanoLC-MS/MS on an Ultimate 3000 system coupled to an Orbitrap Fusion Tribrid mass spectrometer (Thermo Fisher). Peptides were separated on an Acclaim PepMap C18 analytical column (Thermo Fisher) using an 80 min acetonitrile gradient (1–90%) in 0.1% formic acid at 300 nL/min. Peptides were ionised by nano-electrospray ionisation at 2.2 kV using a stainless-steel emitter (30 μm internal diameter) with a capillary temperature of 275 °C. Data were acquired in DDA mode, with FTMS1 spectra recorded at 120,000 resolution over an m/z range of 400–2000, an AGC target of 400,000, and a maximum injection time of 50 ms. Dynamic exclusion was applied for 30 s (±10 ppm), and MS2 precursor ions were isolated within a 1.6 m/z window. ITMS2 spectra were acquired with an AGC target of 5000 and a maximum injection time of 50 ms. Raw data were processed using Proteome Discoverer v2.1 (Thermo Fisher) and searched against the UniProt human reference proteome (downloaded January 2023; 81,579 sequences) using SEQUEST HT. Precursor mass and MS/MS tolerances were set to 10 ppm and 0.6 Da, respectively. Carbamidomethylation of Cys was set as a fixed modification, while Met oxidation, N-terminal acetylation, and phosphorylation of Ser, Thr, and Tyr were included as variable modifications. Up to two missed tryptic cleavages were permitted, and peptide-spectrum matches were filtered to a 1% FDR.

### 2.14. AlphaFold3 Protein Structure Modelling

The full-length human LMTK3 sequence (UniProt A0A3B3ISL5) was retrieved in FASTA format. Protein structure prediction was performed using the AlphaFold3 server v3.0.1 [65,66,67,68]. Resulting models were visualised and analysed using PyMOL v2.5.7 (Schrödinger, New York, NY, USA).

### 2.15. Statistical Analysis

Unless otherwise stated, all experiments were performed using at least three independent biological replicates. Data were analysed using GraphPad Prism v10.3.1 (GraphPad Software, Boston, MA, USA). Results are presented as mean ± standard error of the mean (SEM), unless otherwise specified. Normality was assessed using the Shapiro–Wilk test prior to parametric analyses. Statistical significance was determined using one-way or two-way analysis of variance (ANOVA), with adjusted (adj.) *p*-values reported where applicable. One-way ANOVA was followed by Tukey’s multiple comparisons post hoc test. Two-way repeated measures ANOVA was performed with the Geisser–Greenhouse correction and followed by Bonferroni’s multiple comparisons test. For two-group comparisons, two-tailed unpaired Student’s *t*-tests were used, with exact *p*-values reported. A *p*-value < 0.05 was considered statistically significant. Significance is annotated as follows: ns (not significant), * *p* < 0.05, ** *p* < 0.01, *** *p* < 0.001, **** *p* < 0.0001.

## 3. Results

### 3.1. Identification of S1258 as a Candidate LMTK3 Phosphorylation Site In Vitro

Intrinsically disordered regions (IDRs) are often subject to phosphorylation, a post-translational modification (PTM) that enhances their functional versatility [69,70,71]. Given the disordered nature of the LMTK3 C-terminus and the established catalytic activity of its kinase domain [41,44,45], we hypothesised that the C-terminal tail of LMTK3 might undergo phosphorylation by the LMTK3-KD, potentially contributing to the regulation of LMTK3 function. To investigate this, we performed *in vitro* kinase assays using [γ-^32^P]-ATP and recombinant LMTK3-KD as the catalytic enzyme (Figures S1A, S2A and S3), with specific C-terminal fragments as substrates. Two fragments corresponding to residues 688–1095 and 1181–1486 were produced for this purpose (Figures S1B, S2B and S3). Construct boundaries were limited by recombinant expression and protein solubility constraints, as intervening linker regions and larger combined fragments suffered from severe aggregation, with only regions obtained in soluble form retained for biochemical analysis.

**Figure 1:**
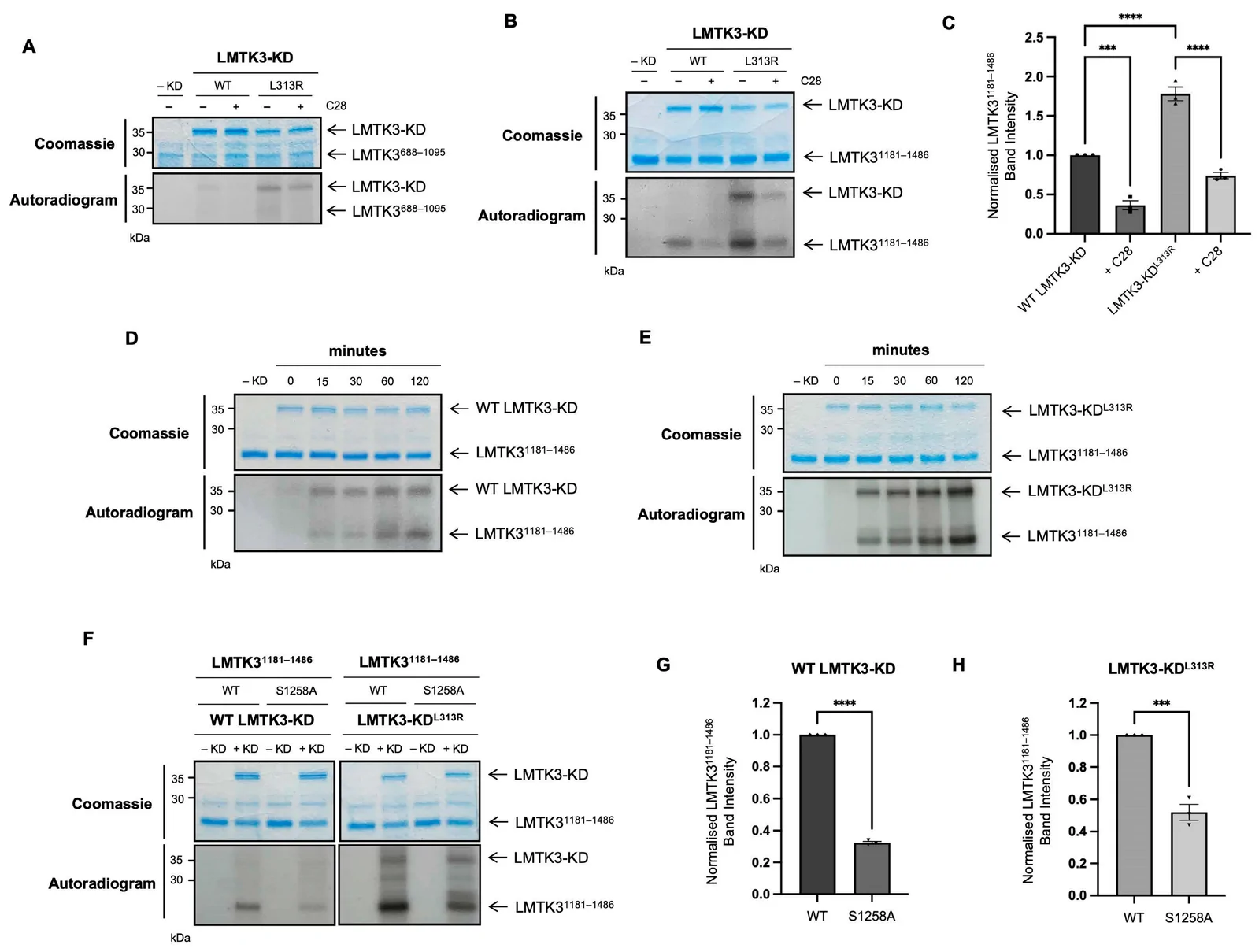
*In vitro* identification of Ser1258 (S1258) as a candidate phosphorylation site within the LMTK3 C-terminus. (**A**,**B**) Kinase assays using LMTK3 C-terminal fragments as substrates: (**A**) residues 688–1095; (**B**) residues 1181–1486. Reactions were performed using wild-type (WT) LMTK3-KD or the constitutively active LMTK3-KD mutant (L313R) in the absence or presence of the LMTK3-specific ATP-competitive inhibitor C28. (**C**) Quantification of LMTK3^1181–1486^ phosphorylation normalised to total substrate protein, from three independent experiments. Data are mean ± SEM; statistical significance was determined by one-way ANOVA with Tukey’s post hoc test. WT LMTK3-KD vs. LMTK3-KD^L313R^, *p* < 0.0001; WT LMTK3-KD vs. WT LMTK3-KD + C28, *p* = 0.0002; LMTK3-KD^L313R^ vs. LMTK3-KD^L313R^ + C28, *p* < 0.0001. (**D**,**E**) Time-course kinase assays (0–120 min) using LMTK3^1181–1486^ as substrate with (**D**) WT LMTK3-KD or (E) LMTK3-KD^L313R^. (**F**) Kinase assays using WT or phospho-null (S1258A) LMTK3^1181–1486^ as substrates with WT LMTK3-KD or LMTK3-KD^L313R^. (**G**,**H**) Quantification of LMTK3^1181–1486^ phosphorylation normalised to total substrate protein, from three independent experiments. Data are mean ± SEM; statistical significance was assessed using a two-tailed unpaired Student’s *t*-test. WT vs. S1258A (**G**) *p* < 0.0001; (**H**) *p* = 0.0006. *** *p* < 0.001, **** *p* < 0.0001.

No phosphorylation was detected in the LMTK3^688–1095^ fragment (Figure 1A), suggesting that this region is not a substrate under these assay conditions. In contrast, both WT LMTK3-KD and the constitutively active LMTK3-KD^L313R^ mutant phosphorylated the LMTK3^1181–1486^ fragment (Figure 1B). The L313R substitution, located immediately following the DYG motif, mimics the oncogenic EGFR L858R mutation, which stabilises the active kinase conformation [41,72]. Consistent with this, LMTK3-KD^L313R^ exhibited enhanced catalytic activity toward the LMTK3^1181–1486^ fragment, as further supported by increased phosphorylation of the known substrate HSP27 (Figure S4). In addition, phosphorylation of the LMTK3^1181–1486^ fragment was significantly reduced by the LMTK3-specific ATP-competitive inhibitor C28 (Figure 1C), confirming that the observed signal is kinase-dependent. Time-course assays also revealed a progressive increase in phosphorylation over time by both WT LMTK3-KD (Figure 1D) and LMTK3-KD^L313R^ (Figure 1E).

To map phosphorylation sites within LMTK3^1181–1486^, we performed LC-MS/MS analysis of the fragment following incubation with WT LMTK3-KD, identifying S1258 as a candidate phosphorylation site (Table S1). Mutation of this residue to Ala significantly reduced phosphate incorporation compared with WT LMTK3^1181–1486^ (Figure 1F, Figures S1C, S2C and S3), as confirmed by densitometric quantification (Figure 1G,H). Additional phosphorylation sites were also detected within the LMTK3^1181–1486^ fragment; however, mutation of the corresponding residues had minimal impact on [γ-^32^P]-ATP incorporation relative to WT LMTK3^1181–1486^ (Figure S5) and were therefore not further analysed. Collectively, these *in vitro* data identify S1258 as a previously uncharacterised candidate phosphorylation site within the LMTK3 C-terminal region and support further investigation of its potential regulatory role.

### 3.2. Introduction of a Phospho-Mimetic Substitution at S1258 Alters Interactions Between LMTK3^1181–1486^ and the Kinase Domain

**Figure 2:**
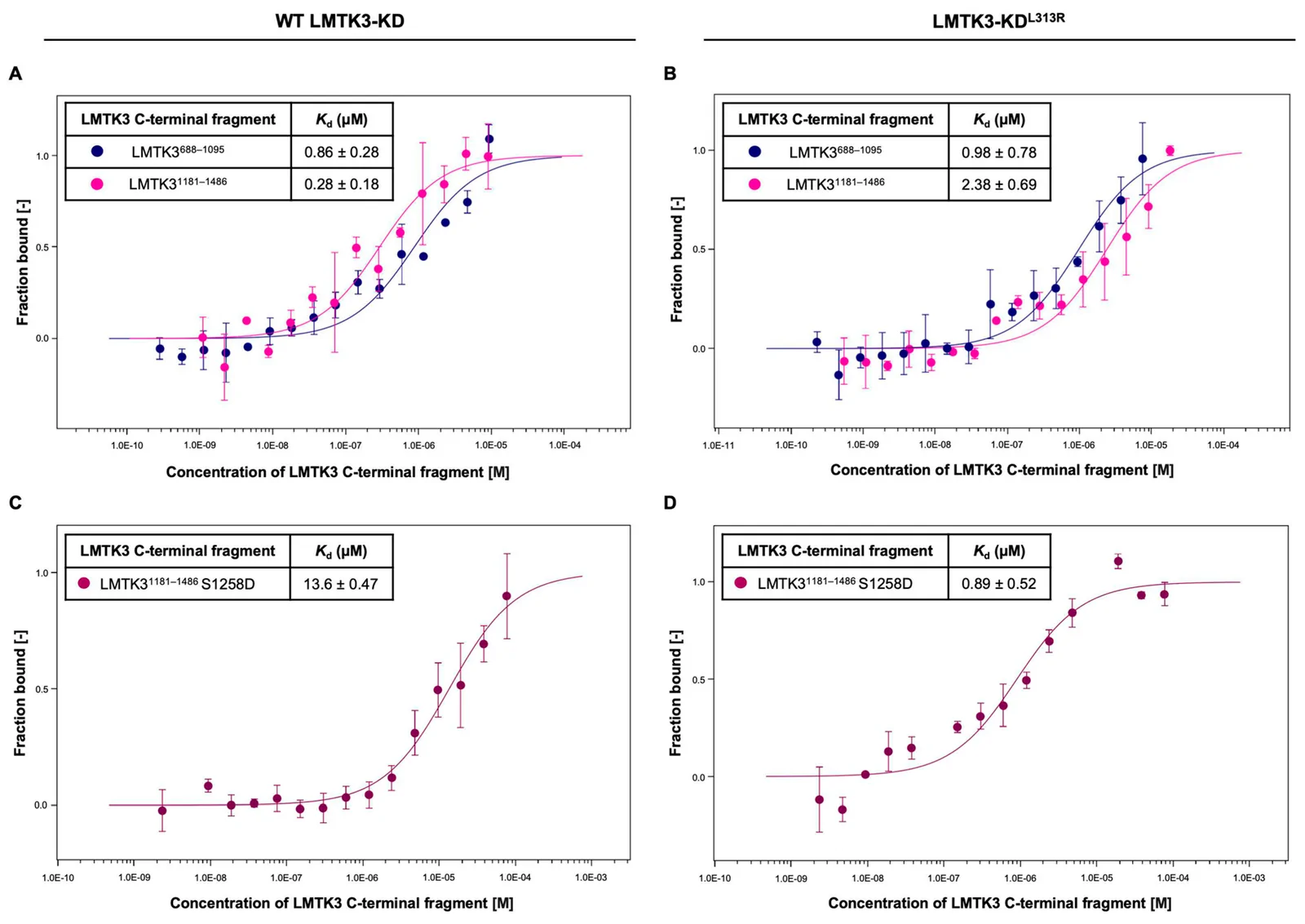
Biophysical analysis of interactions between LMTK3-KD and LMTK3 C-terminal fragments. (**A**–**D**) Microscale thermophoresis (MST) binding assays measuring interactions between fluorescently labelled WT LMTK3-KD (**A**,**C**) or constitutively active LMTK3-KD^L313R^ (**B**,**D**) and LMTK3 C-terminal fragments. (Panels **A**,**B**) show unmodified C-terminal fragments: residues 688–1095 (blue) and 1181–1486 (magenta). (Panels **C**,**D**) show the phospho-mimetic S1258D variant of LMTK3^1181–1486^. Titrations were performed by incubating a constant concentration of labelled kinase domain with increasing concentrations of the indicated C-terminal fragment. Fraction bound was calculated from normalised fluorescence changes relative to curve amplitude and plotted against ligand concentration [M]. Calculated *K*_d_ values were 0.86 ± 0.28 μM and 0.98 ± 0.78 μM for LMTK3^688–1095^ binding to WT LMTK3-KD and LMTK3-KD^L313R^, respectively. Corresponding *K*_d_ values for LMTK3^1181–1486^ were 0.28 ± 0.18 μM and 2.38 ± 0.69 μM, respectively. The phospho-mimetic LMTK3^1181–1486^ S1258D fragment exhibited *K*_d_ values of 13.6 ± 0.47 μM and 0.89 ± 0.52 μM for WT LMTK3-KD and LMTK3-KD^L313R^, respectively. Error bars represent ±1 standard deviation (SD) from at least three independent MST measurements.

Phosphorylation within IDRs can modulate protein interactions, thereby influencing protein conformation and function [73,74,75,76]. Having identified S1258 as a candidate LMTK3 phosphorylation site *in vitro*, we used MST, which detects binding-induced changes in the movement of fluorescently labelled molecules within a temperature gradient [77,78] to investigate interactions between the previously described C-terminal regions and the kinase domain. Since binding was initially assessed using WT LMTK3-KD in the absence of ATP/Mg^2+^, the kinase domain is expected to adopt predominantly the inactive conformation previously described by X-ray crystallography, in which Tyr311 from the DYG motif occupies the ATP-binding site [41]. The LMTK3^688–1095^ fragment bound to WT LMTK3-KD with a dissociation constant (*K*_d_) of 0.86 ± 0.28 μM, while the LMTK3^1181–1486^ fragment exhibited stronger binding (*K*_d_ = 0.28 ± 0.18 μM, Figure 2A and Figure S6). Subsequently, to assess whether kinase activation state influences these binding interactions, we employed the constitutively active L313R mutant as a model for active LMTK3 [41]. Binding of LMTK3-KD^L313R^ to the LMTK3^688–1095^ fragment was comparable to that of WT LMTK3-KD (*K*_d_ = 0.98 ± 0.78 μM), whereas binding to the LMTK3^1181–1486^ fragment was reduced approximately 10-fold, with the *K*_d_ shifting to 2.38 ± 0.69 μM (Figure 2B and Figure S7).

To investigate the potential effect of S1258 phosphorylation on these interactions, we generated a phospho-mimetic (S1258D) mutant of the LMTK3^1181–1486^ fragment, substituting Ser with Asp to mimic the negative charge introduced by phosphorylation (Figures S1C, Figures S2C and S3) [79]. The phospho-mimetic fragment exhibited a 40-fold decrease in binding affinity to WT LMTK3-KD (*K*_d_ = 13.6 ± 0.47 μM) relative to the unmodified C-terminal fragment (Figure 2C and Figure S8), but showed enhanced binding to the constitutively active LMTK3-KD^L313R^ mutant (*K*_d_ = 0.89 ± 0.52 μM, Figure 2D and Figure S9). These findings suggest that phospho-mimetic substitution at S1258 alters interactions between the C-terminal LMTK3^1181–1486^ fragment and the kinase domain, shifting binding preference toward the constitutively active LMTK3-KD^L313R^ mutant while indicating that binding can occur independently of modification at this site.

### 3.3. An AlphaFold3-Predicted α-Helical Motif Contributes to Binding Between LMTK3^1181–1486^ and the Kinase Domain

**Figure 3:**
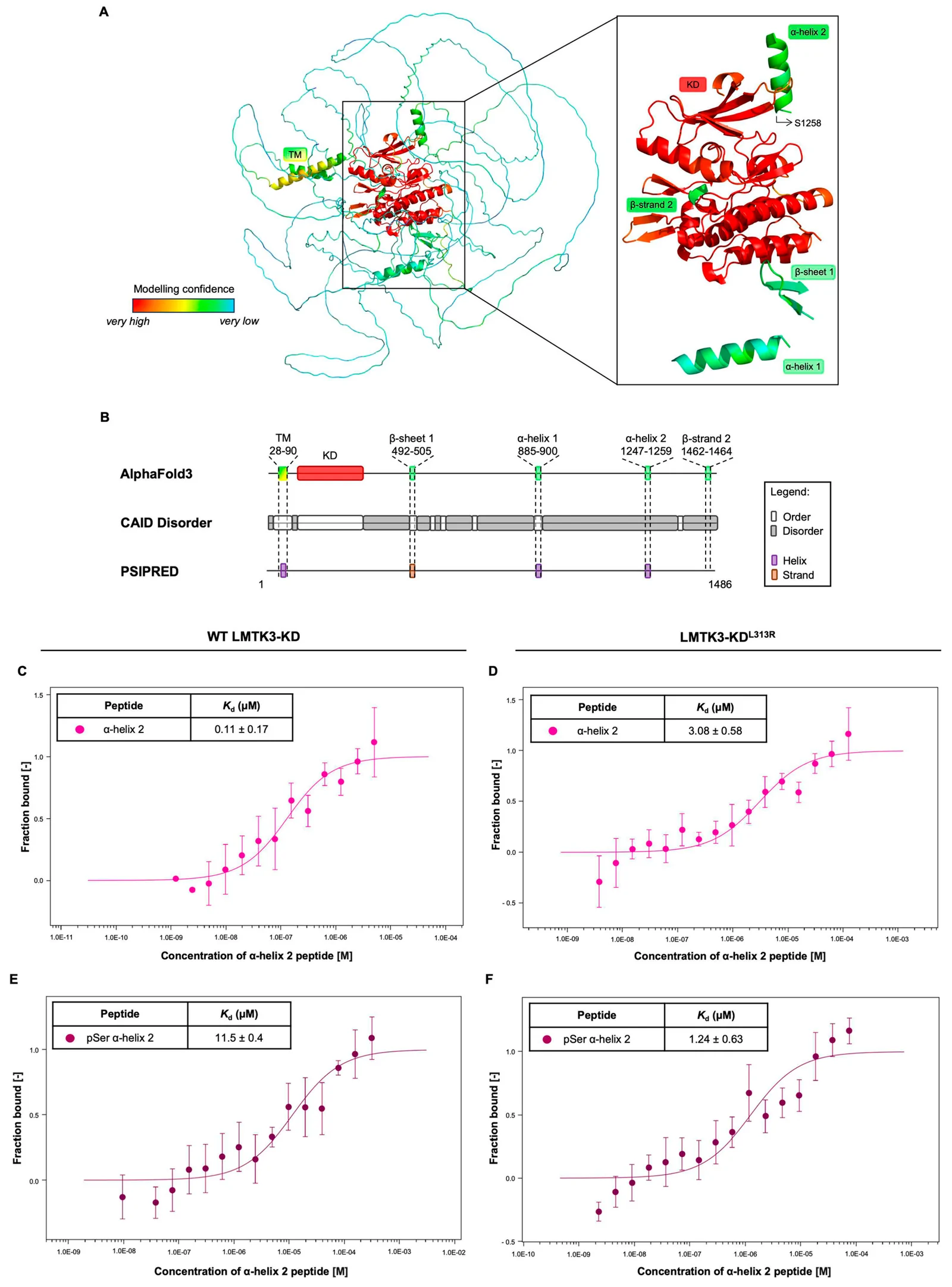
An AlphaFold3-predicted α-helical motif within LMTK3^1181–1486^ contributes to binding with LMTK3-KD. (**A**) AlphaFold3 model of full-length human LMTK3 coloured by per-residue confidence (pLDDT): red, very high (>90); orange, high (70 ≤ pLDDT ≤ 90); green, low (50 ≤ pLDDT < 70); blue, very low (<50). N-terminal transmembrane region, spanning residues 28–90, is indicated as TM. Boxed region highlights the kinase domain and predicted structured elements within the C-terminus. Right: Magnified view of the boxed region with disordered regions omitted for clarity. Predicted structural features are indicated: kinase domain (KD, residues 134–444), α-helix 1 (885–900), α-helix 2 (1247–1258), β-sheet 1 (492–505), and β-strand 2 (1462–1464). (**B**) Schematic representation of predicted structural features across the LMTK3 sequence. Top: AlphaFold3-predicted motifs, coloured as in (**A**). Middle: CAID disorder prediction profile, with grey and white regions indicating disorder and order, respectively. Bottom: PSIPRED secondary structure prediction highlighting regions corresponding to AlphaFold3-predicted α-helices and β-strands. Dashed lines indicate the sequence positions of AlphaFold3-predicted structural elements across the CAID and PSIPRED profiles. (**C**–**F**) MST binding assays measuring interactions between fluorescently labelled WT LMTK3-KD (**C**,**E**) or LMTK3-KD^L313R^ (**D**,**F**) and the α-helix 2 peptide. (Panels **C**,**D**) show the unmodified peptide, while (Panels **E**,**F**) show the phosphorylated (pSer) α-helix 2 variant. Titrations were performed by incubating a constant concentration of labelled kinase domain with increasing concentrations of peptide. Fraction bound was calculated from normalised fluorescence changes relative to curve amplitude and plotted against ligand concentration [M]. Calculated *K*_d_ values for the unmodified α-helix 2 peptide were 0.11 ± 0.17 μM and 3.08 ± 0.58 μM for WT LMTK3-KD and LMTK3-KD^L313R^, respectively. Corresponding *K*_d_ values for the phosphorylated α-helix 2 were 11.5 ± 0.4 μM and 1.24 ± 0.63 μM, respectively. Error bars represent ±1 SD from at least three independent MST measurements.

Short, ordered motifs within IDRs frequently act as functional sites mediating intra- or intermolecular contacts, including regulatory interactions in kinases [80,81,82]. To determine whether such motifs are present within the LMTK3 C-terminal region, we generated a full-length LMTK3 model using AlphaFold3, based on the 1486-residue UniProt sequence (A0A3B3ISL5) [65,66,67,68]. This model recapitulated the canonical bilobal kinase fold [41] and predicted that the C-terminus of LMTK3 is predominantly disordered, adopting an extended random coil conformation (Figure 3A). The predicted aligned error (PAE) map also indicated lower confidence in the positioning of this region relative to the kinase domain (Figure S10), consistent with a flexible, dynamic tail [66,83]. Within this predominantly disordered region, AlphaFold3 identified several short segments of secondary structure that exhibited higher confidence in their spatial positioning than the surrounding disordered regions of the C-terminus (Figure 3A and Figure S10), including two α-helices (α-helix 1, residues 885–900, TEQLLMSLREDVTRNL; α-helix 2, 1247–1258, SEQIKARLSRLS), a two-stranded β-sheet (492–505, LTVTESSRGLNLEC), and a short β-strand (1462–1464, VSP). Disorder and secondary-structure predictions were consistent with the AlphaFold3 model, supporting the presence of these predicted structural elements within the largely disordered C-terminus (Figure 3B) [84,85,86].

Notably, S1258 is positioned within the predicted α-helix 2, implicating this element as a candidate interaction site between LMTK3^1181–1486^ and the kinase domain. To test this, peptides corresponding to the α-helix 2 sequence in both unmodified (QGNSEQIKARLSRLSLALPPLTLTP) and phosphorylated forms (QGNSEQIKARLSRL[pSer]LALPPLTLTP) were synthesised, and their binding to LMTK3-KD was assessed using MST. The unmodified α-helix 2 peptide recapitulated the binding behaviour of the LMTK3^1181–1486^ fragment, showing high affinity for WT LMTK3-KD and reduced binding to the constitutively active LMTK3-KD^L313R^ mutant (*K*_d_ = 0.11 ± 0.17 μM and 3.08 ± 0.58 μM, respectively; Figure 3C,D, Figures S11 and S12). In contrast, the phosphorylated α-helix 2 peptide displayed a reversed binding pattern, consistent with the phospho-mimetic (S1258D) fragment, showing reduced affinity for WT LMTK3-KD and increased binding to LMTK3-KD^L313R^ (*K*_d_ = 11.5 ± 0.4 μM and 1.24 ± 0.63 μM, respectively; Figure 3E,F, Figures S13 and S14). Together, these results suggest that α-helix 2 represents a primary interaction site within the LMTK3^1181–1486^ sequence for binding to LMTK3-KD.

### 3.4. S1258A Mutation Attenuates LMTK3-Dependent Upregulation of ERα and Alters Oncogenic Phenotypes in Breast Cancer Cells

**Figure 4:**
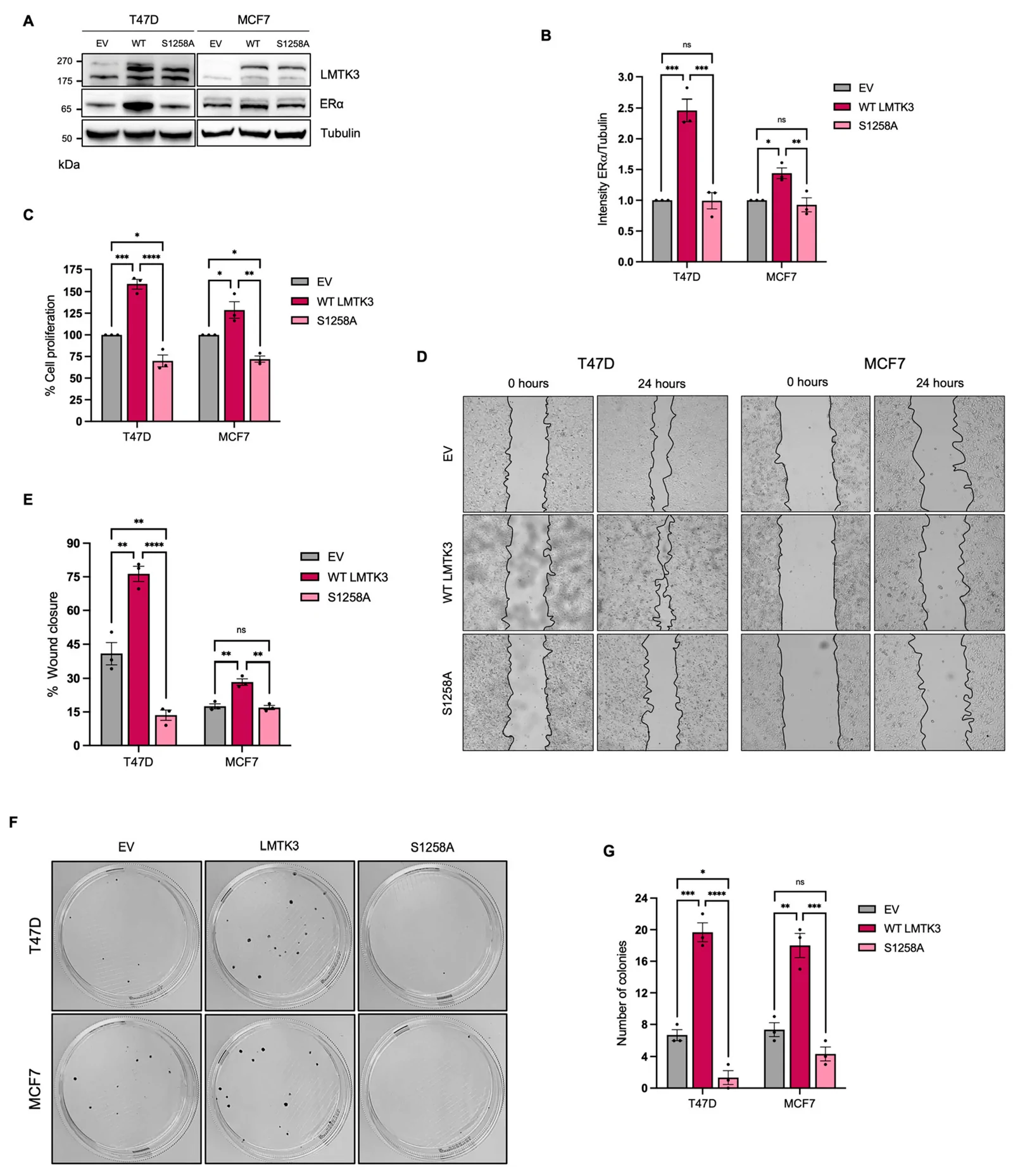
Effects of LMTK3 S1258A on ERα regulation, proliferation, migration, and colony formation in breast cancer cells. (**A**) Western blot analysis of ERα expression in T47D and MCF7 cells stably overexpressing WT LMTK3, the phospho-null S1258A mutant, or an empty vector (EV) control. Tubulin was used as a loading control. (**B**) Quantification of ERα protein levels normalised to EV control, from three independent experiments. Data are mean ± SEM; statistical significance was determined by one-way ANOVA with Tukey’s post hoc test. T47D, EV vs. WT LMTK3, *p* = 0.0005; EV vs. S1258A, *p* = 0.997; WT LMTK3 vs. S1258A, *p* = 0.0004. MCF7, EV vs. WT LMTK3, *p* = 0.0154; EV vs. S1258A, *p* = 0.7155; WT LMTK3 vs. S1258A, *p* = 0.0067. (**C**) Cell proliferation assessed by crystal violet staining after 96 h, normalised to EV control, from three independent experiments. Data are mean ± SEM; statistical significance was determined by one-way ANOVA with Tukey’s post hoc test. T47D, EV vs. WT LMTK3, *p* = 0.0004; EV vs. S1258A, *p* = 0.0124; WT LMTK3 vs. S1258A, *p* < 0.0001. MCF7, EV vs. WT LMTK3, *p* = 0.0312; EV vs. S1258A, *p* = 0.035; WT LMTK3 vs. S1258A, *p* = 0.0012. (**D**) Representative images of wound closure at 0 h and 24 h corresponding to (**E**). (**E**) Cell migration assessed by wound-healing assay and quantified as percentage wound closure after 24 h, from three independent experiments. Data are mean ± SEM; statistical significance was determined by one-way ANOVA with Tukey’s post hoc test. T47D, EV vs. WT LMTK3, *p* = 0.0012; EV vs. S1258A, *p* = 0.0046; WT LMTK3 vs. S1258A, *p* < 0.0001. MCF7, EV vs. WT LMTK3, *p* = 0.0015; EV vs. S1258A, *p* = 0.9385; WT LMTK3 vs. S1258A, *p* = 0.0012. (**F**) Representative images of colonies corresponding to (**G**). (**G**) Colony formation assay assessing clonogenic survival over 21 days, from three independent experiments. Data are mean ± SEM; statistical significance was determined by one-way ANOVA with Tukey’s post hoc test. T47D, EV vs. WT LMTK3, *p* = 0.0002; EV vs. S1258A, *p* = 0.0285; WT LMTK3 vs. S1258A, *p* < 0.0001. MCF7, EV vs. WT LMTK3, *p* = 0.0014; EV vs. S1258A, *p* = 0.2291; WT LMTK3 vs. S1258A, *p* = 0.0004. * *p* < 0.05, ** *p* < 0.01, *** *p* < 0.001, **** *p* < 0.0001, ns: not significant.

To investigate the functional relevance of S1258, we generated stable ER-positive human BC cell lines (T47D and MCF7) constitutively overexpressing full-length WT LMTK3, the phospho-null (S1258A) mutant, or an empty vector (EV) control. Consistent with previous reports that LMTK3 stabilises ERα and enhances its transcriptional activity [45], WT LMTK3 overexpression increased ERα protein levels relative to EV controls (Figure 4A,B). Notably, S1258A failed to elevate ERα expression, with protein levels remaining comparable to EV controls in both cell lines.

We next assessed the impact of S1258A on established LMTK3-driven oncogenic phenotypes [33]. As expected, WT LMTK3 promoted cell proliferation (Figure 4C), migration (Figure 4D,E), and clonogenic growth (Figure 4F,G) in T47D and MCF7 cells [38,45]. In contrast, S1258A overexpression significantly suppressed proliferation relative to WT and EV groups in both cell lines (Figure 4C). Similarly, S1258A attenuated migration relative to WT LMTK3, with migratory activity in T47D cells also reduced below EV control levels (Figure 4D,E). Colony formation assays further demonstrated that S1258A-overexpressing T47D cells displayed reduced clonogenic potential compared with EV controls, whereas clonogenicity in MCF7 cells was comparable between S1258A and EV groups (Figure 4F,G).

Collectively, these results suggest that the S1258A mutation disrupts LMTK3-mediated ERα stabilisation and impairs proliferation, migration, and clonogenic survival in ER-positive human BC cell lines [33,38,45]. These findings therefore support a regulatory role for S1258 within the LMTK3 C-terminus and implicate this residue in LMTK3-driven oncogenic signalling, warranting further investigation.

### 3.5. TMT-Based Proteomic and Phosphoproteomic Analysis Indicates Alterations in Cell-Cycle Progression, DNA Damage Response, and Extracellular Matrix Organisation Following S1258A Mutation

**Figure 5:**
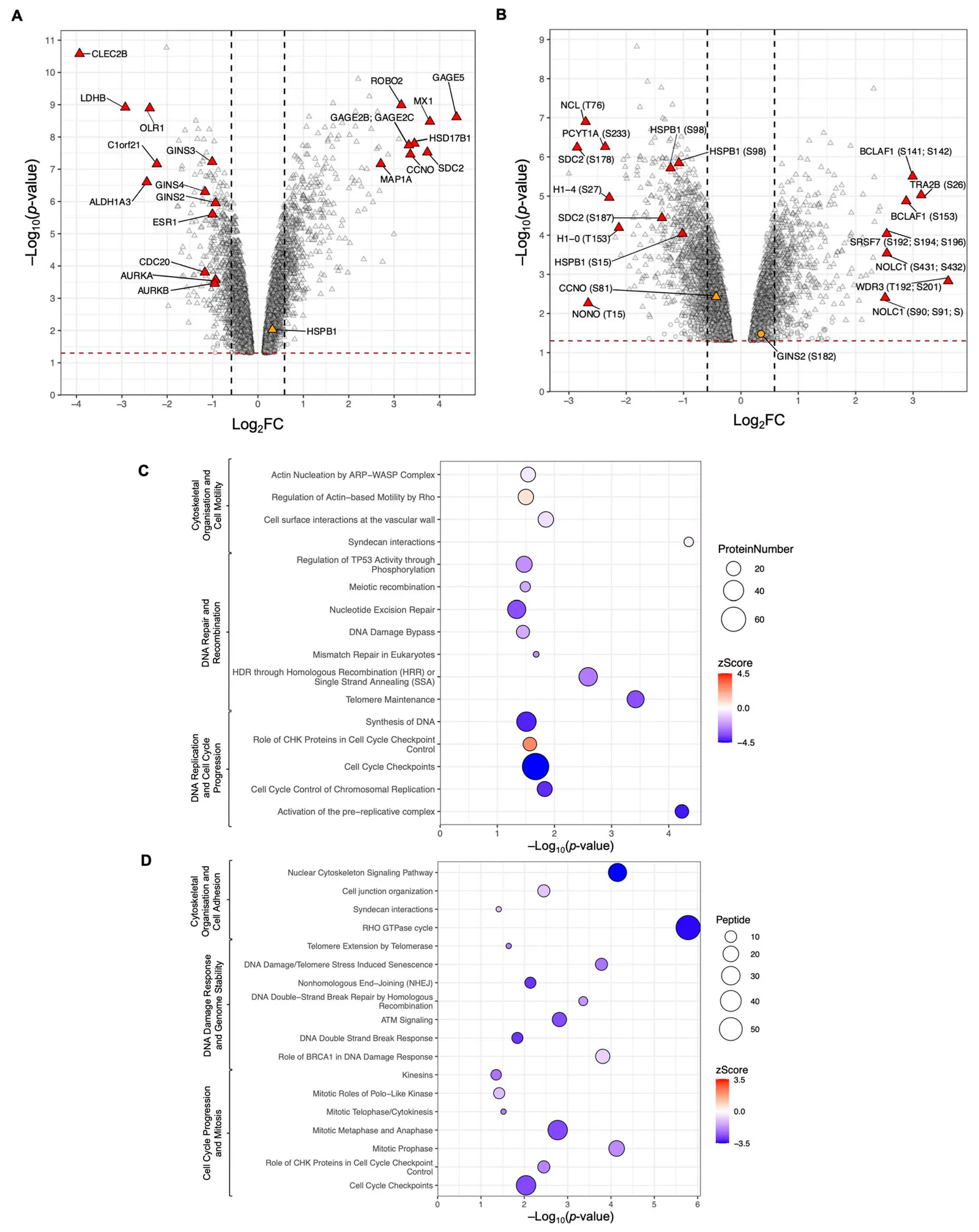
Proteomic and phosphoproteomic changes following S1258A mutation highlight alterations in cell-cycle progression, DNA damage response, and extracellular matrix organisation. (**A**,**B**) Volcano plots of differentially expressed proteins (**A**) and phosphopeptides (**B**) following S1258A mutation in LMTK3-expressing T47D cells (S1258A vs. WT). Log_2_FC is plotted against −Log_10_ (raw *p*-value). The red horizontal dashed line indicates the significance threshold (raw *p* = 0.05), while the black vertical dashed lines represent Log_2_FC cut-offs of ± 0.585, corresponding to a 1.5-fold change in linear scale. Selected targets are highlighted in orange when |Log_2_FC| < 0.585 and in red when |Log_2_FC| ≥ 0.585; all other features are shown in grey. Data points passing the false discovery rate (FDR) threshold (adj. *p* < 0.05) are shown as triangles, whereas points failing the FDR threshold are shown as circles. (**C**,**D**) Ingenuity Canonical Pathway Analysis of significantly altered proteins (**C**) and phosphopeptides (**D**). Pathways are grouped by biological function and displayed with corresponding −Log_10_(*p*-values). z-scores indicate predicted activation states based on expression or phosphorylation changes; negative scores (in blue) indicate predicted pathway inhibition in S1258A mutant cells relative to WT LMTK3. Pathways shown were selected based on statistical significance and biological relevance to the observed cellular phenotypes. Circle sizes represent the number of identified proteins or peptides that map to that particular pathway.

To assess molecular changes associated with the altered proliferative, migratory, and clonogenic phenotypes observed in S1258A mutant cells, we performed quantitative TMT-based proteomic and phosphoproteomic profiling of WT LMTK3 and S1258A-expressing T47D cells (Table S2). Global proteomic analysis identified 8403 proteins, of which 3439 were significantly altered between conditions (adj. *p* < 0.05; Figure 5A). Among these, 897 proteins exhibited changes in abundance greater than 1.5-fold (|Log_2_FC| > 0.58), including 506 upregulated and 391 downregulated proteins. Phosphoproteomic analysis identified 10,068 phosphopeptides, of which 2884 were differentially regulated (adj. *p* < 0.05; Figure 5B), corresponding to 830 unique proteins.

Ingenuity Pathway Analysis (IPA) on significantly altered proteins (Table S3) and phosphorylation sites (Table S4) identified biological processes related to cell-cycle progression, DNA damage response, and extracellular matrix (ECM) organisation (Figure 5C,D) [63]. Notably, pathways implicated in DNA replication initiation, DNA synthesis, cell-cycle checkpoint control, and mitotic progression were predicted to be inhibited based on negative z-scores.

At the protein level, Syndecan-2 (SDC2), a key component of the ECM, was upregulated (Figure 5A), while phosphorylation at residues linked to SDC2 function (S178 and S187) was reduced (Figure 5B) [87], potentially reflecting altered regulation of cell–matrix and cell–cell adhesion. Cyclin O abundance was also increased (Figure 5A), with decreased phosphorylation at S81 (Figure 5B), a modification required for CDK2-dependent kinase activity [88]. Given the role of CDK2 in the G1/S transition and S-phase progression [4], reduced S81 phosphorylation is consistent with altered CDK2-dependent cell-cycle regulation. Core components of the GINS complex, including GINS2, GINS3, and GINS4 (Figure 5A) [89], were downregulated, with reduced phosphorylation detected at GINS2 S182 (Figure 5B). The GINS complex is essential for CMG (Cdc45-MCM-GINS) helicase activity during DNA replication initiation, and its downregulation is consistent with reduced replication fork progression and increased cell cycle arrest [90]. In addition, AURKA, AURKB, and Cdc20, proteins involved in maintaining genomic stability by directing several mitotic events, including chromosome alignment and mitotic exit, were downregulated (Figure 5A) [91,92]. Collectively, these findings indicate that the S1258A mutation is associated with significant changes in protein abundance and phosphorylation across biological processes linked to cell-cycle progression, DNA damage response, and ECM organisation.

### 3.6. LMTK3 S1258A Reduces Tumour Growth in Breast Cancer Mouse Models

**Figure 6:**
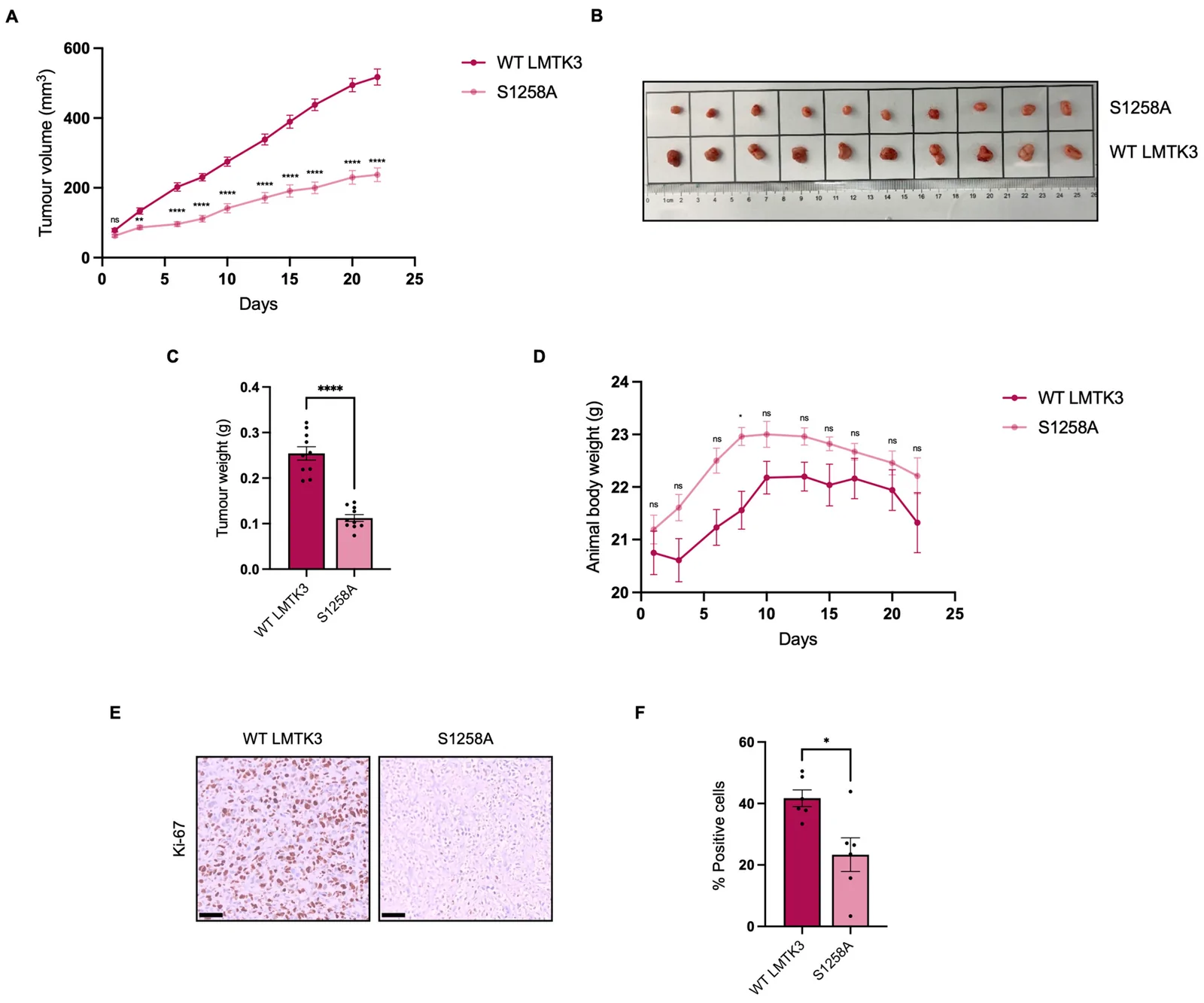
LMTK3 S1258A reduces tumour growth *in vivo*. (**A**) Tumour growth curves showing tumour volume (mm^3^) in mice xenografted with MCF7 cells stably overexpressing WT LMTK3 or the phospho-null S1258A mutant (*n* = 10 per group). Data are mean ± SEM; statistical significance was determined by two-way repeated measures ANOVA with Bonferroni’s multiple comparisons test. Day 1, *p* = 0.3448; Day 3, *p* = 0.008; Day 6–22, *p* < 0.0001. The mean difference in tumour volume between groups was 156.5 mm^3^ (95% CI: 122.1–191.0 mm^3^). (**B**) Representative images of excised tumours collected at endpoint corresponding to (**C**). (**C**) Endpoint tumour weights (g) from WT LMTK3 or S1258A xenograft groups. Data are mean ± SEM; statistical significance was assessed using a two-tailed unpaired Student’s *t*-test (*p* < 0.0001). The mean difference in tumour weight between groups was 0.1418 g (95% CI: 0.1070–0.1766 g). (**D**) Animal body weight (g) over time in WT LMTK3 or S1258A xenograft groups. Data are mean ± SEM; statistical significance was determined by two-way repeated measures ANOVA with Bonferroni’s multiple comparisons test. No sustained differences in body weight were observed between groups during the study. The mean difference in animal weight between groups was 0.8391 g (95% CI: 0.07649–1.602). (**E**) Representative images of Ki-67 immunohistochemistry corresponding to (**F**). (**F**) Quantification of proliferative index, defined as the percentage of Ki-67-positive nuclei relative to total nuclei, in excised MCF7 tumours from WT LMTK3 or S1258A groups at endpoint (*n* = 6 per group). Scale bar = 50 μm. Data are mean ± SEM; statistical significance was determined by two-tailed unpaired Student’s *t*-test (*p* = 0.0189). The mean difference in proliferative index between groups was 18.37 (95% CI: 4.727–32.02). * *p* < 0.05, ** *p* < 0.01, **** *p* < 0.0001, ns: not significant.

To investigate the functional role of S1258 *in vivo*, MCF7 cells stably overexpressing WT LMTK3 or the phospho-null S1258A mutant were injected into the fourth mammary fat pad of female BALB/c nude mice (*n* = 10 per group), and tumour growth was monitored for 22 days (Table S5). Tumours derived from S1258A-overexpressing MCF7 cells exhibited significantly reduced growth, with lower tumour volumes throughout the study (Figure 6A) and reduced final tumour weights (Figure 6B,C) compared with tumours expressing WT LMTK3. Notably, no consistent differences in body weight were observed between groups during the study (Figure 6D), suggesting no evidence of systemic toxicity. Furthermore, immunohistochemical analysis revealed reduced Ki-67 staining in LMTK3 S1258A mutant tumours compared with WT LMTK3, providing additional evidence of reduced BC cell proliferation in S1258A mutant MCF7 xenografts, which is consistent with the observed impairment in tumour growth (Figure 6E,F; Table S5).

Collectively, these findings indicate that the S1258A mutation attenuates the tumour-promoting function of LMTK3 *in vivo*, consistent with our *in vitro* observations, and further support a regulatory role for S1258 in LMTK3-driven breast cancer progression.

## 4. Discussion

LMTK3 is a key oncogenic kinase implicated in several cancer types, particularly BC, where it promotes tumour progression, invasiveness, metastasis, and therapy resistance [33,38,39,40,41,42,43,44,45,46]. Despite its clinical relevance and potential as a therapeutic target [36,37,47,48,49,50,51,52,53], the molecular mechanisms regulating LMTK3 function remain poorly understood, particularly within its extended, Pro-rich C-terminal region, which is predicted to be intrinsically disordered [32,33,34]. IDRs frequently harbour short, linear motifs that function as regulatory elements by mediating intra- and intermolecular interactions or undergoing context-dependent folding upon binding [80,81]. In protein kinases, such motifs can modulate conformational dynamics, provide docking sites for regulatory proteins, or act as sites of regulatory phosphorylation [82]. These properties make the extended LMTK3 C-terminal IDR a compelling candidate for regulating kinase function.

**Figure 7:**
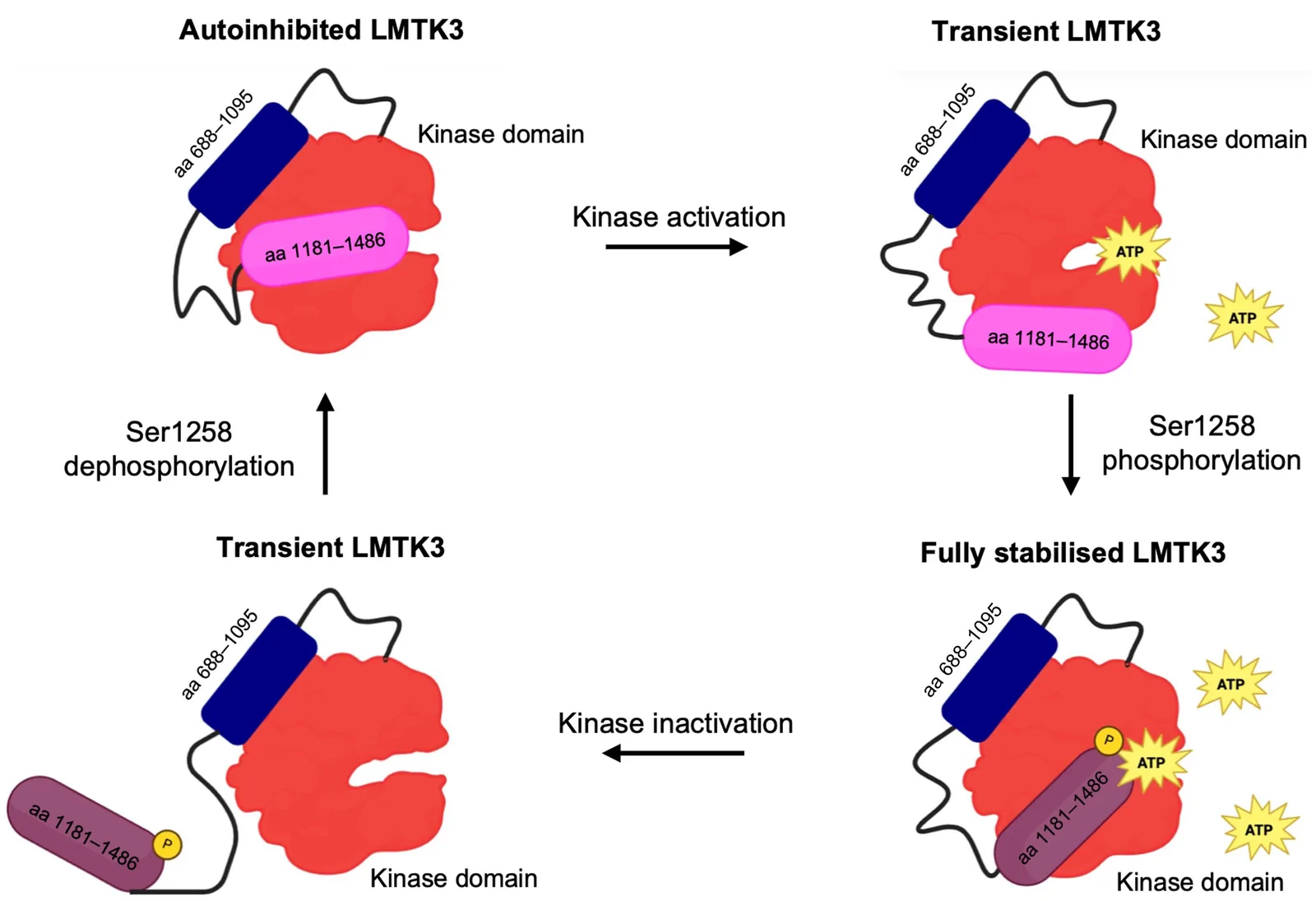
Proposed model for regulation of LMTK3 by S1258 phosphorylation. In a putative inactive kinase state, the unmodified LMTK3^1181–1486^ region is proposed to engage in regulatory interactions with the kinase domain, potentially mediated in part by the α-helix 2 motif encompassing S1258, consistent with a closed regulatory conformation. Upon kinase activation, these interactions may be weakened, resulting in reduced binding of the unmodified C-terminal region to the active kinase domain conformation. Phosphorylation of S1258 within the α-helix 2 motif is proposed to further modulate these interactions and favour binding to the active kinase domain conformation. Following kinase inactivation, the phosphorylated LMTK3^1181–1486^ region is proposed to exhibit reduced affinity for the WT LMTK3-KD, consistent with phosphorylation favouring interactions with the active kinase state. Whether S1258 phosphorylation contributes to LMTK3 activation, occurs as a consequence of activation, or directly influences catalytic activity remains to be determined. Functionally, the S1258A mutation is associated with altered LMTK3-dependent oncogenic phenotypes, including ERα upregulation, proliferation, migration, clonogenic survival, and tumour growth.

Consistent with this hypothesis, we identified α-helix 2 (residues 1247–1258) within the LMTK3^1181–1486^ fragment as a candidate regulatory element that interacts with the kinase domain, although contributions from surrounding disordered regions cannot be excluded. The preferential binding of both α-helix 2 and the LMTK3^1181–1486^ fragment to WT LMTK3-KD, relative to the constitutively active L313R mutant, supports a model in which this region contributes to stabilisation of an autoinhibited kinase conformation by potentially restricting the conformational flexibility required for activation (Figure 7). Similar autoinhibitory mechanisms have been described in other kinases, where flexible N- or C-terminal regions engage the kinase domain to regulate kinase function, as exemplified by Abl and PKC family members [93,94,95].

Within α-helix 2, we identified S1258 as a candidate phosphorylation site *in vitro*, consistent with previous reports that the closely related family member LMTK2 undergoes autophosphorylation [96,97]. Moreover, S1258 lies within a sequence compatible with the reported LMTK3 substrate motif [41], supporting its potential physiological relevance. Notably, introduction of the phospho-mimetic S1258D mutation altered binding between the LMTK3^1181–1486^ fragment and the kinase domain, reducing affinity for WT LMTK3-KD while enhancing binding to the constitutively active L313R mutant (Figure 7). Collectively, these MST binding profile comparisons support a model in which S1258 modification functions as an active-state conformational switch. Whereas the unmodified LMTK3^1181–1486^ fragment preferentially engages the inactive kinase state, introduction of a negative charge at S1258 reverses this preference, favouring interaction with the active kinase conformation. Such behaviour is consistent with established roles of phosphorylation within IDRs, where modification of individual residues can remodel protein interactions and regulate protein function [73,74,75,76].

The functional importance of S1258 was supported by the observation that the phospho-null S1258A mutation attenuated multiple oncogenic phenotypes in ER-positive BC cell lines, including proliferation, migration, and clonogenic survival. Reduced tumour burden and decreased Ki-67 staining in orthotopic S1258A MCF7 xenografts further demonstrated that disruption of this regulatory mechanism probably impairs LMTK3 kinase activity and therefore LMTK3-driven tumour progression *in vivo*. However, we do not directly show LMTK3 activation by S1258 phosphorylation. Proteomic and phosphoproteomic profiling further implicated pathways involved in DNA replication, cell-cycle progression, DNA damage response, and cell adhesion signalling [63], with replication initiation and checkpoint control predicted to be inhibited. Consistent with these pathway-level changes, components of the GINS complex and mitotic regulators, including AURKA, AURKB, and Cdc20, were differentially expressed [89,90,91,92]. As dysregulation of these proteins has previously been linked to impaired cancer cell proliferation and tumour progression [98,99,100,101], these molecular changes provide a plausible explanation for the reduced oncogenic phenotypes observed in S1258A-expressing cells.

Although we identify S1258 as a potential regulatory switch, several limitations should nevertheless be considered when interpreting these findings. The highly disordered nature of the full-length LMTK3 C-terminal region (residues 688–1486) presented challenges for recombinant expression as a soluble protein suitable for characterisation, necessitating the use of shorter soluble fragments for biophysical and biochemical studies. Although this strategy enabled robust quantitative binding measurements, it may not fully capture higher-order conformational dynamics that contribute to regulation of the intact C-terminal tail. In addition, while S1258A substitution markedly attenuated [γ-^32^P]-ATP incorporation, residual radiolabel signal was observed, suggesting that S1258 is unlikely to represent the sole phosphorylation site within the C-terminal region. Consistent with this interpretation, LC-MS/MS analysis identified additional candidate phosphorylation sites, raising the possibility of cooperative, hierarchical, or processive phosphorylation within the intrinsically disordered C-terminal tail. Defining the relative contribution of these sites will require future studies employing combinatorial Ala mutants.

Although S1258 was robustly identified as a phosphorylation site *in vitro*, endogenous phosphorylation at this residue remains to be confirmed. Future studies using pS1258-specific antibodies or targeted endogenous mass spectrometry will be required to determine whether S1258 is phosphorylated under physiological conditions, while complementary biochemical approaches will be necessary to identify the upstream regulatory enzymes responsible for its dynamic regulation. Although no kinase or phosphatase has yet been directly linked to LMTK3 S1258 regulation, previous studies demonstrating CDK5/p35- and Src-dependent phosphorylation of other LMTK family members [32,102,103,104,105], as well as interactions between LMTK proteins and phosphatase complexes such as PP1/inhibitor-2 [97] suggest potential regulatory mechanisms that warrant further investigation.

Our global proteomic and phosphoproteomic datasets likewise provide mechanistic hypotheses rather than definitive molecular mechanisms. Although they identify coordinated alterations in pathways associated with DNA replication, mitosis, and cell-cycle regulation, they do not distinguish direct LMTK3-regulated effectors from secondary consequences of altered signalling. Furthermore, individual candidate hits were not orthogonally validated by Western blotting in the current work. Future targeted validation will therefore be required to define the direct molecular effectors of S1258-dependent LMTK3 regulation and clarify their contribution to the observed cellular phenotypes.

While our functional and *in vivo* validation focused on ER-positive BC models, reflecting the well-established role of LMTK3 in ERα stabilisation [45], LMTK3 overexpression is also widely reported in triple-negative BC [36], as well as in non-mammary malignancies, including colorectal, gastric, non-small cell lung, and bladder cancers [47,48,49,50,51]. Given that the C-terminal IDR is a conserved structural feature of LMTK3, it is plausible that the phosphorylation-dependent regulatory mechanism described here extends beyond ERα-dependent signalling. Evaluating whether disruption of this C-terminal switch similarly attenuates oncogenic signalling in ER-negative BC and other solid tumours therefore represents an important direction for future investigation.

Finally, although our data support a model in which S1258 phosphorylation regulates kinase domain interactions, the relationship between this conformational switch and catalytic activity remains unresolved. However, the fact that S1258A reduces tumour growth suggests that S1258 phosphorylation may activate LMTK3. Future biochemical studies evaluating the kinase kinetics of a combined L313R/S1258A double mutant will be important to determine whether S1258 phosphorylation is a consequence of kinase activation or instead drives catalytic activation. Likewise, defining whether the downstream phenotypes arise from altered kinase activity, altered scaffolding interactions, or a combination of both—and establishing the contribution of additional intra- or intermolecular interactions—will be essential for fully understanding the regulatory role of the LMTK3 C-terminal region.

## 5. Conclusions

Collectively, this study identifies α-helix 2 as a previously uncharacterised regulatory element within the LMTK3 C-terminal tail, provides evidence that this motif contributes to interactions with the kinase domain, and supports a model in which phosphorylation at S1258 functions as a regulatory molecular switch. Disruption of this switch through S1258A mutation impaired LMTK3-driven oncogenic phenotypes in BC cell and xenograft models, further highlighting the functional importance of this region in tumour progression. More broadly, these findings expand our understanding of LMTK3 regulation by revealing a functional role for its intrinsically disordered C-terminal region and illustrate how PTMs within disordered regions can modulate kinase behaviour and downstream signalling. Together, this work establishes the LMTK3 C-terminus as an important regulatory interface with potential implications for targeting LMTK3-driven BC progression.

## Figures and Tables

**Table 1:** Summary of MST conditions for LMTK3 target-ligand interactions.

Ligand	Ligand Concentration Range	Fluorescent Target	Target Concentration
LMTK3^688–1095^	9.37 μM to 0.29 nM	WT LMTK3-KD	30 nM
LMTK3^1181–1486^	9 μM to 1.1 nM	WT LMTK3-KD	30 nM
LMTK3^1181–1486^ S1258D	77 μM to 2.35 nM	WT LMTK3-KD	30 nM
LMTK3^688–1095^	7.5 μM to 0.23 nM	LMTK3-KD^L313R^	20 nM
LMTK3^1181–1486^	18 μM to 0.55 nM	LMTK3-KD^L313R^	20 nM
LMTK3^1181–1486^ S1258D	77 μM to 2.35 nM	LMTK3-KD^L313R^	20 nM
QGNSEQIKARLSRLSLALPPLTLTP	5 μM to 1.22 nM	WT LMTK3-KD	30 nM
QGNSEQIKARLSRL(pSer)LALPPLTLTP	313 μM to 9.54 nM	WT LMTK3-KD	30 nM
QGNSEQIKARLSRLSLALPPLTLTP	125 μM to 3.81 nM	LMTK3-KD^L313R^	50 nM
QGNSEQIKARLSRL(pSer)LALPPLTLTP	75 μM to 2.29 nM	LMTK3-KD^L313R^	50 nM

pSer denotes phosphoserine.

## Data Availability

The original contributions presented in this study are included in the article/Supplementary Materials. Further inquiries can be directed to the corresponding author(s).

## Supplementary Materials

The following supporting information can be downloaded at: https://www.mdpi.com/article/10.3390/cells15161473/s1. Data supporting the purification and validation of LMTK3 constructs are provided in Supplementary Figures S1–S3. Data supporting the increased catalytic activity of the LMTK3-KD^L313R^ mutant and analysis of candidate phosphorylation sites within LMTK3^1181–1486^, respectively, are provided in Supplementary Figures S4 and S5. MST raw data quality assessments for LMTK3-KD interactions with C-terminal fragments and α-helix 2 peptides are provided in Supplementary Figures S6–S9 and S11–S14. The predicted aligned error (PAE) map for the full-length LMTK3 AlphaFold3 model is provided in Supplementary Figure S10. Data supporting identification of the LMTK3 S1258 phosphorylation site *in vitro* are provided in Supplementary Table S1. Protein- and phosphoproteomic analyses of T47D cells are provided in Supplementary Table S2. Protein- and phosphoproteomic Ingenuity Pathway Analysis (IPA) enrichment results are provided in Supplementary Tables S3 and S4, respectively. Individual tumour volume measurements and Ki-67 immunohistochemical quantification from MCF7 xenografts are provided in Supplementary Table S5.
